# Permeability of Phospholipid Membranes to Divalent Cations: The Effect of Pulsed Electric Field

**DOI:** 10.3390/molecules31010151

**Published:** 2026-01-01

**Authors:** Małgorzata Jurak, Monika Sujka, Agnieszka Ewa Wiącek, Urszula Pankiewicz

**Affiliations:** 1 Department of Interfacial Phenomena, Institute of Chemical Sciences, Faculty of Chemistry, Maria Curie-Skłodowska University in Lublin, Maria Curie-Skłodowska Sq. 3, 20-031 Lublin, Poland; agnieszka.wiacek@mail.umcs.pl; 2 Department of Analysis and Food Quality Assessment, Faculty of Food Science and Biotechnology, University of Life Sciences in Lublin, Skromna 8, 20-704 Lublin, Poland; urszula.pankiewicz@up.lublin.pl

**Keywords:** phospholipid, monolayer, liposomes, yeast, divalent cations, pulsed electric field, electroporation

## Abstract

Achieving a high nutritional value of food often involves fortifying microorganisms (such as bacteria and yeast) used in baking and dairy industry with essential elements. The aim of this study was to investigate the effect of a pulsed electric field (PEF) on the penetration and accumulation of Ca^2+^ and Mg^2+^ ions into model membranes of the food-grade yeast *Saccharomyces cerevisiae*. Simplified model membranes (monolayers and liposomes) were constructed using the phospholipid 1-palmitoyl-2-oleoyl-*sn*-glycero-3-phosphatidylcholine (POPC). The Langmuir monolayer technique, dynamic light scattering (DLS) and microelectrophoresis were employed to characterize the physicochemical properties of the model membranes investigated. The results showed significant molecular-level differences in the interactions of the selected cations with lipid monolayers and bilayers in liposome structures. Both cations deeply penetrated the membrane’s hydrophilic region, yet two competing effects were evident: expansion induced by hydrated Mg^2+^ and condensation driven by Ca^2+^ bridging. Furthermore, the application of PEF increased the concentration of ions absorbed by the liposomes. Specifically, optimized PEF parameters resulted in cation accumulation within the model membranes, ranging from 6 to 13%. This finding correlates well with the increased Ca^2+^ and Mg^2+^ uptake observed in real yeast cells, providing a deeper understanding of the cell membrane-environment interface and the underlying processes.

## 1. Introduction

The pulsed electric field (PEF) method is used in food technology to monitor microbiological safety and assess the quality of food products throughout the entire production process [1,2]. In medicine and molecular biology, PEF facilitates the introduction of various chemical compounds, such as proteins, dyes, drugs, or nucleic acids, into cells [3,4]. Short electrical pulses trigger localized rearrangements in the lipid bilayer, leading to the transient appearance of subnanometer hydrophilic pores that close within nanoseconds due to thermal and mechanical fluctuations. This process is called electroporation [5,6]. The treatment modifies the cell membrane so that molecules, which are normally unable to pass through, can now enter the cell. It is effective for a wide range of cells, including bacteria and yeast [7,8,9,10,11,12]. By carefully optimizing the PEF parameters, the membrane’s permeability can be precisely controlled, allowing for the transport of molecules into or out of the cells [13]. Furthermore, selecting the appropriate intensity and duration can cause irreversible electroporation, leading to the complete disintegration of the membrane [2,5]. This feature allows for effective control over the microbiological quality of a product [1].

In living organisms, Ca^2+^ and Mg^2+^ ions are crucial for cellular signaling and metabolism, with their transmembrane transport tightly regulated to maintain homeostasis. Ca^2+^ levels are kept at nanomolar concentrations inside cells and millimolar outside. In contrast, Mg^2+^ ions, a key ATP and enzyme cofactor, have higher intracellular levels than Ca^2+^ ions but are also tightly regulated [14]. Exposure to pulsed electric field (PEF) alters the transport of Ca^2+^ and Mg^2+^ ions across cell membranes. Moderate PEF can depolarize the membrane and activate voltage-gated Ca^2+^ channels, causing transient Ca^2+^ influx and downstream signaling [15,16]. Strong, short pulses induce transient membrane pores, leading to non-selective ion movement—mainly Ca^2+^ entry and some Mg^2+^ loss [5]. Excessive Ca^2+^ accumulation can disrupt homeostasis and deplete ATP levels [17]. Overall, PEF primarily enhance Ca^2+^ influx through channel activation or membrane permeabilization, with indirect effects on Mg^2+^ transport.

This study investigated the effect of PEF on the penetration of Ca^2+^ and Mg^2+^ ions into liposomes. These well-designed phospholipid vesicles can mimic the cell membranes of *Saccharomyces cerevisiae* yeast. The liposomes were prepared via the dry film hydration technique using 1-palmitoyl-2-oleoyl-*sn*-glycero-3-phosphatidylcholine (POPC). POPC is one of the most widely used and well-characterized phospholipids for creating simple model biological membranes [18]. It was chosen because phosphatidylcholine (PC), the class of phospholipid to which POPC belongs, is one of the dominant glycerophospholipids found in the cellular membranes of *S. cerevisiae* [19]. Additionally, the Langmuir monolayer technique was utilized to study the behavior of a single membrane leaflet in the presence of these ions.

The core of this paper involved physicochemical analyses of model cell membrane systems. By comparing these models to natural yeast cells, we attempted to correlate their properties and quantitatively analyzed the penetration and incorporation efficiency of inorganic divalent ions (Ca^2+^ and Mg^2+^) into these structures. The research confirmed the usefulness of PEF in the process of enriching microbial cells, demonstrating that the efficiency is strictly dependent on the field parameters and duration of action. This method holds significant application potential, particularly in food technology. In addition, this research contributes new insights and improves the understanding of the crucial phenomena occurring at the cell membrane-environment interface, which is foundational for developing strategies to encapsulate and release active substances using cellular or liposome carriers.

## 2. Results and Discussion

Numerous studies have shown that divalent cations like Ca^2+^ and Mg^2+^ interact with the headgroups of membrane lipids, significantly influencing the membrane’s physical properties [20,21,22,23,24,25]. Despite their identical charge, these ions possess specific chemical properties (Table 1) that allow them to exert distinct effects on phospholipid membranes. The precise mechanisms underlying these divergent behaviors, however, are not yet fully elucidated.

In this study, we used Langmuir monolayers and liposomes composed of zwitterionic POPC as simplified membrane models of *Saccharomyces cerevisiae*. This approach allowed us to investigate the binding of divalent Ca^2+^ and Mg^2+^ ions, a process relevant to the enrichment of yeast cells with these elements. Experiments were conducted with and without 100 μg/mL of Ca^2+^ or Mg^2+^ ions to determine their influence before and after PEF treatment, which was applied exclusively to the liposomes. The chosen ion concentration was based on the range previously optimized for yeast enrichment [8]. By optimizing PEF parameters, it is possible to control membrane permeability and enable the transport of molecules and ions into or out of the cells. For comparison, the electroporation process was also used to modify the permeability of yeast cell membranes under the same conditions as those utilized for liposomes.

### 2.1. Langmuir Monolayers

**Figure 1:**
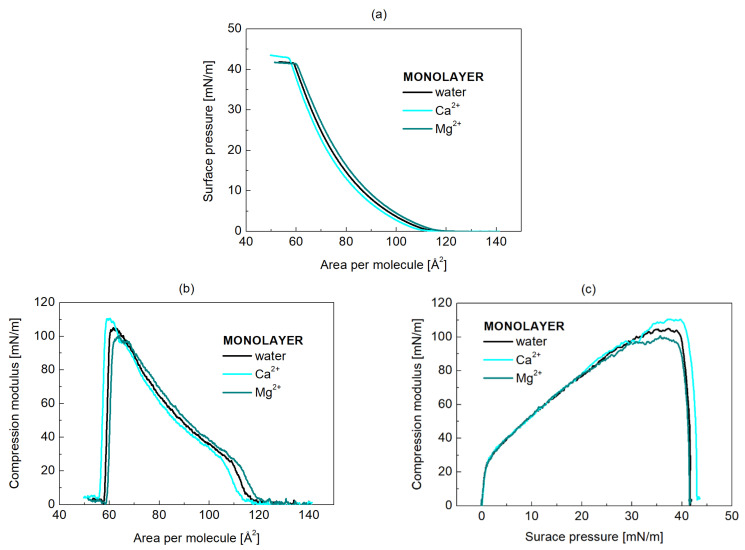
(**a**) Compression isotherms of POPC monolayer on a water subphase at 25 °C in the absence (black) and presence of either Ca^2+^ (cyan) or Mg^2+^ (dark cyan). The isotherms shown are representative of three independent measurements. Compression modulus as a function of (**b**) area per molecule and (**c**) surface pressure is also presented.

Surface pressure versus mean molecular area (π−A) isotherms were recorded for POPC monolayer at the liquid/air interface (Figure 1a). This monolayer serves in the present study as a simplified model for a yeast membrane leaflet. All acquired compression isotherms are shown in Figure S1 (Supplementary Materials). The representative isotherms (Figure 1a) were utilized to calculate the compression modulus ($C_{S}^{-1}$), Equation (1), a critical parameter for characterizing the monolayer’s resistance to compression and thereby its physical state. The values of this parameter allowed for the characterization of the membrane elasticity—specifically, its degree of packing and ordering—at various surface pressures and how these properties were influenced by the presence of cations (Figure 1b,c).

The POPC isotherm (Figure 1a) is typical for unsaturated monolayers [26]. The lift-off area is observed at approximately 112 Å^2^/molecule (Table 2). As the mean molecular area (MMA) decreases, the surface pressure (π) gradually rises to 41.7 mN/m when the monolayer collapses. The shape of the isotherm indicates the liquid-expanded (LE) phase, as defined by Davies and Rideal for monolayers with a compression modulus ($C_{S}^{-1}$) value around 100 mN/m [27]. Adding a divalent cation to the water subphase has a measurable, though minimal, effect at a concentration of 100 µg/mL. Ca^2+^ condenses the films, as evidenced by a shift to a smaller mean molecular area (108.9 Å^2^/molecule), while Mg^2+^ slightly expands the layer at low surface pressures (114.5 Å^2^/molecule). In the presence of Ca^2+^, π increases more sharply, reaching collapse at 42.9 mN/m, indicating tighter molecular packing (Table 2). This is revealed by the increased compression modulus values (110.6 mN/m) and the decreased limiting area ($A_{lim}$, 60.3 Å^2^/molecule). $A_{lim}$ represents the mean molecular area of the most packed monolayer, which is characterized by the maximal value of the compression modulus ($C_{S,\;max}^{-1}$). If $A_{lim}$ is assumed to be the area of a circle with radius r, the intermolecular spacing (D) in the monolayers can be approximately calculated based on 2r. The distance between POPC molecules decreases by 0.10 Å in the presence of Ca^2+^ and increases by 0.16 Å in the presence of Mg^2+^ (Table 2). The reduced average distance between neighboring molecules suggests strong condensation of lipids due to ion bridging [20].

Table 2 lists also the calculated values for $C_{S}^{-1}$ at 30 mN/m ($C_{S,\;30}^{-1}$), as this pressure corresponds to the internal lateral pressure of natural membranes.

Moreover, *in situ* Brewster Angle Microscopy (BAM) was used to determine monolayer thickness (d). An increase in monolayer stiffness was found to correlate with an increase in its height above the water subphase. POPC monolayers showed a clear response to divalent cations: they became thicker with 100 µg/mL Ca^2+^ (from 2.90 nm to 3.26 nm) and thinner with Mg^2+^ (down to 2.49 nm). These shifts in height are consistent with the cation-induced changes in phospholipid tail ordering previously suggested by the compression modulus.

As shown in Table 2, a decrease in the area per lipid corresponds to a reduced intermolecular distance (D), an increase in monolayer thickness (d) and compression modulus ($C_{S}^{-1}$) values pointing out greater ordering of the hydrocarbon chains when Ca^2+^ ions are in the subphase. The effect of Mg^2+^ ions, meanwhile, is the opposite.

To determine the effect of the ions on monolayer stability, we investigated the kinetics of the monolayer’s response to an external force using constant surface pressure (π) experiments. Changes in the relative mean molecular area ($A/A_{i}$) were measured over approximately a five-hour period while maintaining π at 30 mN/m through constant-rate barrier oscillations. $A_{i}$ is defined as the initial area at time zero, corresponding to the point at which the monolayer attains the target surface pressure. As was mentioned early, this specific pressure is equivalent to the internal lateral pressure found in biomembranes and bilayers, suggesting a comparable molecular packing density in the monolayer [28].

**Figure 2:**
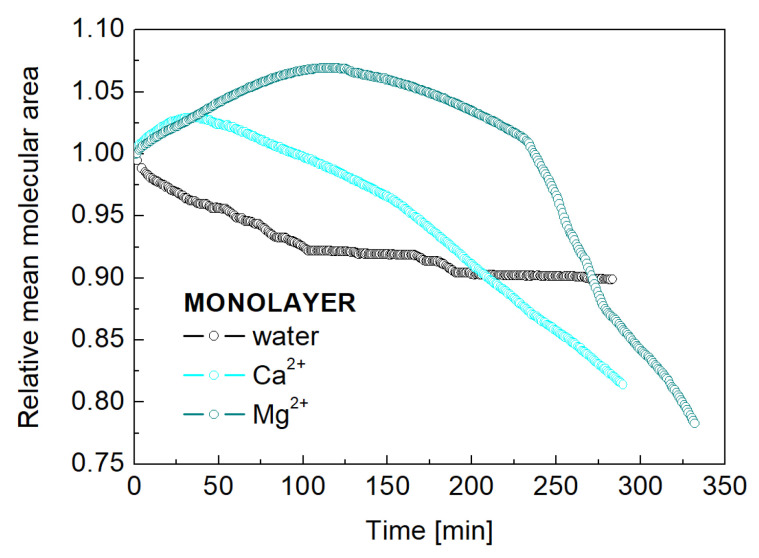
Time-dependent changes in the relative mean area per molecule ($A/A_{i}$) for a Langmuir monolayer of POPC. The monolayer was formed on a subphase containing water, Ca^2+^ or Mg^2+^ ions (100 mg/mL) and compressed to a surface pressure of 30 mN/m.

The data reveal that the mean molecular area of the POPC monolayer on water is not quite stable, showing a 8% reduction after two hours of equilibration. In the presence of cations, monolayer behavior differs (Figure 2). The POPC/Ca^2+^ monolayer first exhibits a 3% area increase (within 40 min) before a 1.5% net reduction is observed after 120 min. The POPC/Mg^2+^ monolayer, however, shows a more gradual increase, peaking at 7% after 120 min, followed by a decrease.

These data indicate that the POPC monolayers have higher stability in the presence of these cations. The initial area increase observed with both ions suggests their penetration into the membrane structure under a constant external pressure. However, the greater packing induced by Ca^2+^ ions appears to cause an earlier loss of monolayer stability, evidenced by the reduction in surface area, which implies molecular desorption. This desorption occurs at a shorter equilibration time compared to the Mg^2+^-containing monolayers. In turn, the increased elasticity provided by Mg^2+^ ions allows the phospholipid molecules to remain in the monolayer longer, as the clear decrease in the area per molecule—demonstrating the loss of molecules from the interface into the bulk phase—occurs later than with Ca^2+^ ions. The difference in the behavior of POPC/Ca^2+^ and POPC/Mg^2+^ monolayers is fundamentally related to how these ions modulate the membrane’s response to external factors in a natural biological systems. In yeast, Ca^2+^ ions act as a crucial secondary messenger. Their influx across the membrane triggers rapid responses to environmental stresses, often by activating specific signaling pathways like the calcineurin pathway. On the other hand, Mg^2+^ ions are a prevalent cofactor essential for stabilizing ATP, DNA, and a vast number of enzymes, contributing to long-term metabolic and structural integrity rather than facilitating a rapid signaling cascade [29]. Findings regarding the behavior of model biological membranes, as well as those made on typical model organisms (e.g., yeast), often allow scientists to identify corresponding biological mechanisms in humans and gain insight into diseases caused by disturbances in ion balance (ion homeostasis).

Taken together, our results demonstrate that Ca^2+^ ions interact with a POPC monolayer differently than do Mg^2+^ ions, leading to distinct structural changes. In a cation-free environment, the phosphocholine polar headgroups of the POPC monolayer align parallel to the subphase [30]. When positively charged Ca^2+^ ions are introduced, they are drawn to the negatively charged phosphate groups, causing the positively charged choline groups to be repelled. This interaction shifts the polar headgroups to a more perpendicular alignment relative to the monolayer’s plane. The result is a more tightly packed lipid arrangement. The structural alterations caused by Mg^2+^ ions are significantly less pronounced than those caused by Ca^2+^ ions. This difference can be attributed to the way these ions are hydrated. In an aqueous solution, both Ca^2+^ and Mg^2+^ are fully hydrated. However, upon approaching the membrane surface, the oxygen atoms within the lipid molecules compete with the water molecules to coordinate with the cations. The stronger interaction between Ca^2+^ and the phosphate groups of the lipids drives the notable structural changes observed.

Based on findings from various studies [20,21,22,23,24,25], the interactions of Ca^2+^ and Mg^2+^ ions with lipid membranes, differ significantly in their effects on hydration, lipid packing, overall membrane structure, and thermodynamics. The relatively low dehydration energy of Ca^2+^ ions (Table 1) allows them to partially shed water. Consequently, they can coordinate directly with the oxygen atoms of the lipid’s phosphate and carbonyl groups [22]. This coordination reduces the effective charge, releasing structured water into the bulk solution, which increases entropy and drives the process as endothermic. In addition, the accumulation of Ca^2+^ ions creates a strong positive field, forcing subsequent ions to highly dehydrate to penetrate the tightly packed headgroup region. Because the lipids in a cation-bound membrane pack more tightly than those in a cation-free membrane, any subsequent incoming, hydrated Ca^2+^ ions must shed more water to penetrate the lipid head region. The resulting loss of electrostatic screening enables a stronger, enthalpy-favored interaction, leading to an exothermic process. The released water, now polarized by the strong field, becomes more ordered, decreasing entropy [22].

Delving deeper into the stabilization mechanism, Ca^2+^ ions can also form bridges between neighboring POPC molecules. This bridging (condensation) effect pulls the lipid molecules closer and restricts their mobility [24], thus acting as a membrane-stabilizing agent. The ability of Ca^2+^ ions to penetrate into the C=O region and directly bind to carbonyl oxygen, despite its larger ionic radius, further highlights its strong membrane binding affinity.

In contrast, Mg^2+^ ions have higher dehydration energy (Table 1) and remain fully hydrated when binding. They interact more weakly with the lipids, and their binding is a simple exothermic process driven by enthalpy, without the complex entropic contributions seen with Ca^2+^ ions [22]. Instead of forming direct bonds, Mg^2+^ ions interact with the lipids through water molecules, acting as a water bridge. These hydrated Mg^2+^ ions primarily reside above the choline groups and are more evenly distributed in the solvent. The repulsion between these charged lipid-ion pairs causes the membrane to expand, leading to a less compact structure. These results provide a crucial understanding of thermodynamics of the adsorption process governing the cellular function of these ions.

The distinct mechanisms by which Ca^2+^ and Mg^2+^ ions interact with phospholipids can be associated with their complementary physiological roles in human cell membranes. Ca^2+^ ions function primarily in rapid signal transduction, where fluctuations in its concentration across the membrane initiate, e.g., neurotransmitter release and muscle contraction [31]. Furthermore, extracellular Ca^2+^ binding helps to maintain plasma membrane integrity and reduce permeability. In contrast, Mg^2+^ ions support the cell’s long-term metabolic stability and efficient transport functions. The synergy between Mg^2+^ and Ca^2+^ ions is essential for the proper functioning of some organs, such as the heart and muscles [32].

### 2.2. Liposomes

#### 2.2.1. Particle Size Distribution and Polydispersity

**Figure 3:**
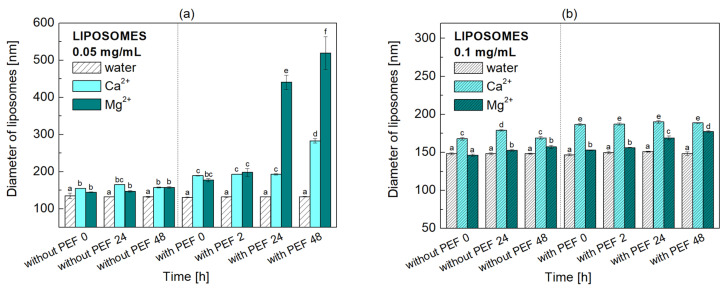
Time-dependent changes in the liposome size (mean hydrodynamic diameter, nm) of POPC dispersions in water, Ca^2+^ or Mg^2+^ solutions, (**a**) 0.05 mg/mL and (**b**) 0.1 mg/mL, as determined by dynamic light scattering (DLS). Error bars denote ± SD. Statistical analysis was performed using one-way ANOVA followed by Tukey’s HSD post hoc test. Different letters above the bars indicate statistically significant differences within each liposome concentration among all tested media and time points (*p* < 0.05).

Divalent cations also act as modifiers of the interaction between phospholipid vesicles. Figure 3 presents the time-dependent changes in the mean hydrodynamic diameter of POPC liposomes obtained by DLS. The initial phospholipid concentrations (before calibration) were 0.05 mg/mL (Figure 3a) and 0.1 mg/mL (Figure 3b). Electroporation experiments were conducted using liposome dispersions that had been allowed to stabilize for 24 h after their initial preparation (“without PEF 24”). Results marked “with PEF 0” were obtained immediately after exposure to the electric field. The error bars represent the standard deviations (SD) of the data set, based on at least three independent measurements.

Control (POPC/water) exhibits homogeneous size distribution with a single sharp peak, having a mean diameter of 134 nm (Figure 3a, Table 3). The homogeneity of the system is confirmed by low values of the polydispersity index (PDI), 0.198. The dispersion remains stable over time, with the liposome size maintained after 24 h (132 nm, PDI = 0.172). At higher POPC concentration (0.1 mg/mL), the liposome diameter is larger (148 nm), and the dispersion is even more homogeneous (PDI < 0.160) and stable (Figure 3b, Table 4). This outcome suggests that at higher POPC concentration, the greater available surface area of the phospholipid film or dispersed phospholipid facilitates the formation of larger vesicles with lower curvature, a state that is thermodynamically favorable once a critical size is reached [33]. Due to POPC being a zwitterionic phospholipid with a net neutral charge, the physical stability of the liposomes it forms is likely maintained by a hydration layer and/or steric effects. These mechanisms can generate repulsive forces that prevent liposome aggregation and fusion.

The addition of metal cations (100 μg/mL) led to a noticeable increase in diameter. Specifically, Ca^2+^ ions resulted in a diameter of 155 nm with a PDI of 0.093, while Mg^2+^ ions yielded a diameter of 145 nm with a PDI of 0.098. Their diameters increased only slightly after 24 h (165 nm for Ca^2+^ and 147 nm for Mg^2+^), with PDIs not greater than 0.121. A two-fold increase in phospholipid concentration resulted in a larger liposome size (179 nm for Ca^2+^ and 153 nm for Mg^2+^) while maintaining the high homogeneity indicated by a very low PDI value (0.061–0.132). The observed changes confirm both the binding of metal cations to the liposomes and the long-term homogeneity and stability of all liposome dispersions. Divalent cations can influence the elastic properties and spontaneous curvature of the lipid bilayer by interacting with the lipid headgroups. This interaction changes the effective headgroup size and shape relative to the hydrophobic tails, which, in turn, can affect liposome size and morphology [34]. By binding to lipid headgroups, divalent cations increase the membrane’s positive surface charge. A sufficiently high net charge generates strong electrostatic repulsion between opposing membrane layers, which is crucial for separating them and stabilizing the final structure of large unilamellar vesicles [34].

Regarding the influence of osmotic pressure, the liposomes in these studies were not maintained under strictly isotonic conditions, which raises the possibility of osmotic imbalance. However, despite this, the addition of Ca^2+^ or Mg^2+^ ions did not cause a noticeable reduction in liposome diameter (Figure 3a,b). Theoretically, osmotic pressure disturbances should result in internal water loss, leading to visible liposome shrinkage. Since this process was not observed, it was concluded that osmotic stress induced by the added ions can be neglected, and that the ion concentration employed (100 μg/mL) is too low to induce significant osmotic effects. The long-term stability of the liposome diameter, with little change after 24 and 48 h, further supports the lack of significant time-dependent osmotic effects. Consequently, the observed increase in particle diameter must be attributed mainly to the aforementioned electrostatic interactions and structural effects, such as ion adsorption or aggregation, rather than volumetric changes caused by osmosis.

PEF treatment did not result in significant changes in the size of POPC liposomes when they were suspended in water at both concentrations. However, the presence of cations led to moderate and comparable size increases immediately after treatment at 0.05 mg/mL, 189 nm for Ca^2+^ and 177 nm for Mg^2+^, with greater PDI, 0.202 and 0.240, respectively. Despite the progressive increase in liposome size, the PDI values remained below or very close to 0.2, indicating a relatively homogeneous dispersion. Over time, both cations induced extensive growth of the hydrodynamic diameter of liposomes, with this effect becoming particularly apparent after 24 h for Mg^2+^ (440 nm/PDI 0.640) and 48 h for Ca^2+^ (283 nm/PDI 0.394). An increase in the hydrodynamic size of liposomes is caused by the aggregation of smaller vesicles into larger structures. This process is primarily driven by three related factors: ion absorption into the liposomes, the subsequent decrease in electrostatic stabilization, and/or a bridging effect where multivalent cations physically link adjacent membranes [35].

On the other hand, dispersions with higher lipid content (0.1 mg/mL) are more stable. Although PEF induces an increase in liposome size, especially for dispersions containing Ca^2+^ ions (187 nm), the diameter is maintained over time with a polydispersity index (PDI) of less than 0.175. A significant increase in liposome size occurs in the presence of Mg^2+^ ions after 24 h of PEF treatment (169 nm/PDI 0.222). The electrostatic and hydration repulsive forces and steric effects are responsible for preventing the aggregation of the positively charged liposomes. Therefore, it is concluded that the increased size is not due to liposome aggregation, but rather to ion adsorption on the membrane and their subsequent penetration into the liposomes. The stability of liposomes is essential for preserving the quality of the substances they encapsulate. The PEF treatment did not disrupt the structural integrity (well-organization) of the liposomes.

Furthermore, the probable creation of transient hydrophilic pores across the bilayers facilitated the penetration of ions into the vesicles, which accounted for the observed increase in average size and also increases the attractiveness of this type of systems in many different applications. One application that may merit particular attention is the use of these systems to study the encapsulation process in living cells, particularly in *Saccharomyces cerevisiae*. A wide range of food-related bioactive compounds has already been successfully encapsulated within yeast cells, including vitamin C [36], vitamin D3 [37], terpenes (carvacrol, eugenol, thymol and geraniol) [38], chlorogenic acid [39], or *Zataria multiflora* Boiss essential oil [40]. Despite considerable progress, the efficiency of yeast cells as delivery systems remains constrained by several intrinsic factors, e.g., the limited porosity of the yeast cell wall and the restricted intracellular volume. These limitations can be alleviated through pretreatment strategies, such as exposure to a pulsed electric field prior to encapsulation. According to Nowosad et al. [41], pretreatment of yeast with PEF (1000 V, with a pulse width of 10 µs, treatment duration of 20 min, frequency of 1 Hz, and 1200 pulses) resulted in approximately a 1.5-fold increase in vitamin C accumulation compared to untreated cells. A comparable effect was observed for vitamin B12, which was co-introduced into the yeast cells together with iron. Application of optimized PEF parameters (exposure time of 20 min, a pulse width of 10 μs, voltage of 1500 V, a number of pulses 1200 and field frequency 1 Hz) led to a 1.4-fold increase in the intracellular concentration of this vitamin within the yeast biomass [42]. On the other hand, PEF pretreatment of yeast cells had no significant effect on the final encapsulation load of oregano essential oil [43]. The aforementioned studies collectively demonstrate that investigations into the impact of pulsed electric field treatment on the efficiency of bioactive compound encapsulation are inherently labour- and time-intensive, as process parameters must be optimized for each specific case. The integration of modelling approaches could substantially streamline and facilitate this optimization process, enabling more efficient application of PEF in yeast-based delivery systems.

#### 2.2.2. Zeta Potential

**Figure 4:**
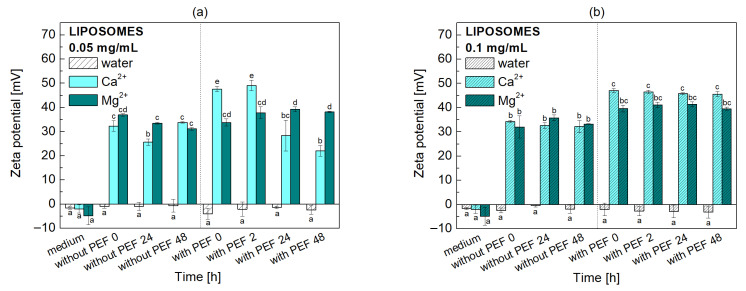
Time-dependent changes in the zeta potential of POPC liposome dispersions in water, Ca^2+^ or Mg^2+^ solutions, (**a**) 0.05 mg/mL and (**b**) 0.1 mg/mL, as determined by microelectrophoresis. Error bars denote ± SD. Statistical analysis was performed using one-way ANOVA followed by Tukey’s HSD post hoc test. Different letters above the bars indicate statistically significant differences within each liposome concentration among all tested media and time points (*p* < 0.05).

Figure 4 presents the zeta potential values determined before and after PEF treatment for the POPC liposomes at two phospholipid concentrations (0.05 mg/mL and 0.1 mg/mL), both in the presence and absence of cations.

The zeta potential was determined from the Smoluchowski equation, based on the measured electrophoretic mobility of the liposomes. This calculation required the simultaneous measurement of the electrical conductivity of the dispersion medium (Figure S2a,b). As expected, the introduction of Ca^2+^ and Mg^2+^ ions increased the bulk conductivity. Notably, the medium containing Mg^2+^ ions exhibited substantially higher conductivity than that with Ca^2+^ ions, primarily because Mg^2+^ ions are more mobile charge carriers. The presence of zwitterionic POPC liposomes, which have very low intrinsic conductivity, exerted a negligible effect on the overall dispersion conductivity, which remained primarily governed by the mobility of the dissolved ions. Although liposome-divalent ion interactions could potentially alter the effective concentration and mobility of the ions, and thus indirectly affect conductivity, this contribution was not statistically significant. Moreover, changes in dispersion conductivity measured over time, both before and after the application of PEF, were also determined to be statistically non-significant (Figure S2a,b). In contrast to the stable conductivity, essential differences were observed in the electrophoretic mobility values of the liposomes following PEF treatment (Figure S3a,b). The changes exhibited an analogous trend in the derived zeta potential values. Therefore, the subsequent discussion will focus predominantly on analyzing the changes observed in the zeta potential.

The zeta potential of liposomes made from the zwitterionic POPC in pure water is a slightly negative value, close to zero [44]. Although POPC molecules are neutral overall at physiological pH, this slight negativity arises from two main factors. First, the orientation of the POPC head group on the liposome surface is usually not perfectly symmetrical. The negatively charged phosphate group (${PO}_{4}^{-}$) tends to be positioned slightly outward from the lipid bilayer, while the positively charged choline group (${N(CH_{3})}_{3}^{+}$) is more buried. This arrangement creates a slight net negative potential at the slipping plane, where, according to current theoretical assumptions, the zeta potential occurs. Second, the liposome’s surface can attract and adsorb a small number of anions from the surrounding water, particularly hydroxyl ions (${OH}^{-}$). This adsorption further contributes to the overall negative charge of the liposome surface. This small, negative zeta potential is highly sensitive to the surrounding environment. Liposome properties are modified when interacting with dissolved ions [44]. The adsorption of cations leads to an elevation in the vesicles’ net surface potential, as confirmed by measurements of electrophoretic mobility and the obtained zeta potential values (32 mV/34 mV for POPC/Ca^2+^ and 37 mV/32 mV for POPC/Mg^2+^, at 0.05 mg/mL and 0.1 mg/mL, respectively). The binding of metal cations to the negatively charged sites on phosphatidylcholine polar heads, due to the electrostatic forces, masks these negative charges. This results in an increase in the overall membrane surface charge, going from weakly negative to positive. Conversely, chloride anions do not adsorb to the membrane but are instead retained within the aqueous phase [45].

The effect of Mg^2+^ is more pronounced than that of Ca^2+^, resulting in a greater increase in the zeta potential. These changes indicate that the metal ions are binding to the liposomes, thereby increasing the positive charge on the membranes. A resulting net charge that is sufficiently high generates strong electrostatic repulsion between membrane surfaces, promoting their separation and stabilizing the liposomes [34]. The observed differences between Ca^2+^ and Mg^2+^ are significant. While electrostatic attraction is the primary driver of these ion-liposome interactions, the specific chemical properties of the metals and the lipid structure also play a role.

On the other hand, PEF treatment has minimal impact on the zeta potential of liposomes in pure water. However, in the presence of cations, a temporary increase in zeta potential is observed within the first two hours after PEF application. This effect is more pronounced with Ca^2+^ (ca. 49 mV at lower POPC concentration). This initial increase is followed by a decrease in zeta potential at the 24 h (28 mV) and 48 h (22 mV) marks. The proportions of charges introduced into the system and the potential charges in the functional groups of phospholipids vary depending on concentration. Therefore, during the equilibrium process, there is initially an excess of positive charges in the double layer, which manifests itself as a high positive zeta potential. After 24 h, the situation stabilizes, and the electrokinetic potential decreases.

Molecular dynamics simulations and X-ray diffraction studies prove that Ca^2+^ and Mg^2+^ interact differently with the lipid bilayer [20]. Ca^2+^ penetrates closer to the glycerol backbone of the lipid, allowing it to form dipole–dipole interactions with the phosphate and carbonyl groups. In contrast, Mg^2+^ remains in the outer region of the lipid head group. This difference is attributed to the higher level of hydration and stronger hydration shell of Mg^2+^. The tightly bound water molecules around Mg^2+^ reduce its affinity for the phosphate and carbonyl groups, explaining why Ca^2+^ has a greater effect on the zeta potential. With time, the potential values decrease continuously by the reduced density of positive charge. The dispersion of higher lipid concentration (Figure 4b), when treated with PEF, exhibits a stable zeta potential over time. The values oscillate between 45 mV and 47 mV for the POPC/Ca^2+^ system and between 39 mV and 41 mV for the POPC/Mg^2+^ system. Zeta potential values are commonly used to assess the stability of colloidal systems and particles of various sizes, among them biological active systems. Based on literature data [46], absolute potential values of 25–30 mV are considered to guarantee system stability regarding electrostatically stabilized systems, e.g., phospholipid-based systems.

The inherent amphiphilic structure of the lipid bilayer renders membranes highly impermeable to ions, while large electrostatic and hydration repulsive forces prevent spontaneous fusion. However, when liposomes are subjected to PEF, a voltage (an electric potential difference) builds across the membrane, causing the formation of transient nanoscale pores. This pore creation dramatically enhances the exchange of ions and solutes across the membrane [47]. Moreover, PEF attenuates ion hydration by disrupting the orientation of water molecules within the ion’s hydration shell [48]. This action results in a reduced ion-water interaction energy and decreased water residence times in the vicinity of the ion. Consequently, the overall strength of the hydration shell diminishes, which enhances ion mobility within the solution. This weakened hydration is pertinent to ion transport across membranes, potentially facilitating the permeation of ions through induced pores in the liposomes, whereas the hydration shell of the chloride ion (Cl^−^) is more resistant to external electric fields because of the distinct ways water molecules reorient around anions versus cations. Ions acting as structure-makers enhance order of the water’s hydrogen-bonding network, while structure-breakers disrupt this order [48].

Additionally, electroporation can also drive membrane electrofusion of closely situated vesicles to form hybrid fusion products. Once the electric field is removed, the edge tension (the energy per unit length of the pore circumference) promotes the rapid closure of these transient pores [47]. This mechanism can be used in many practical applications. In recent years, PEF technology has been successfully applied to enrich *Saccharomyces cerevisiae* cells with essential mineral ions such as Ca^2+^, Mg^2+^, Zn^2+^, or Fe^3+^. According to studies by Pankiewicz et al. [7,8] and Nowosad et al. [10], controlled electroporation using moderate field strengths (typically 0.7–3 kV/cm, 10 µs pulses) temporarily increased cell membrane permeability, facilitating ion uptake without compromising yeast viability. Under optimized conditions, PEF-treated cultures accumulated up to 3.5-fold more Ca^2+^, 2-fold more Mg^2+^ and Zn^2+^, and 2.6-fold more Fe^3+^ compared to untreated controls. Such mineral-enriched yeast biomass can be used as natural mineral supplements, ingredients in functional foods, or components of fortified bakery and fermentation products, offering an alternative to inorganic mineral salts with improved absorption and biological compatibility.

### 2.3. Penetration of Ions into Liposomes and Yeast Cells

It is well known that membrane charging and subsequent pore formation are highly determined by the size of an electroporated particle [49]. Liposomes (typically 50–300 nm) exhibit significantly faster membrane charging kinetics than yeast cells (5–10 µm) and thus can require different pulse durations and amplitudes to reach the same induced transmembrane voltage. Additionally, electroporation response of lipid vesicles and living cells is influenced by differences in membrane composition, intracellular/extravesicular conductivity, and pore resealing kinetics. In the current literature, there are no universally accepted threshold values of PEF parameters required to induce electroporation in liposomes. Existing reports demonstrate a broad range of effective pulse parameters, from nanosecond high-intensity pulses (MV/m) used to induce controlled release from liposomes, to micro- and millisecond pulses with lower field strengths [50]. This variability further confirms the absence of a universal “minimal” electroporation condition for lipid vesicles. In our experiment, we had to assume certain starting parameters, so we decided to apply PEF conditions that were previously optimized for yeast [8].

**Figure 5:**
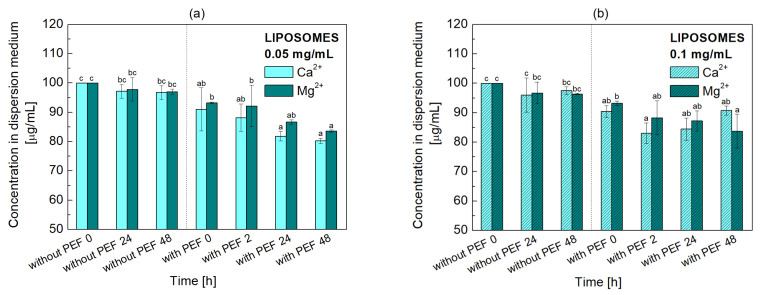
Concentration of Ca^2+^ and Mg^2+^ ions in the medium of POPC liposome dispersions, (**a**) 0.05 mg/mL and (**b**) 0.1 mg/mL, both before and after exposure to a pulsed electric field (PEF). Error bars denote ± SD. Statistical analysis was performed using one-way ANOVA followed by Tukey’s HSD post hoc test. Different letters above the bars indicate statistically significant differences within each liposome concentration among all tested media and time points (*p* < 0.05).

**Figure 6:**
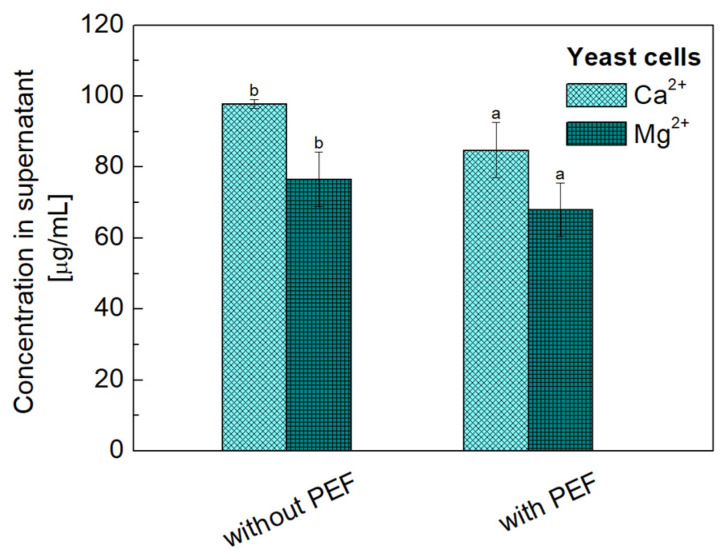
Effect of PEF on concentration of Ca^2+^ and Mg^2+^ ions in solution with suspended *Saccharomyces cerevisiae* cells. Initial ions concentration was 100 µg/mL. Error bars denote ± SD. Different letters above bars for the same ion (Ca^2+^ or Mg^2+^) indicate statistically significant differences at *p* < 0.05 by Tukey HSD test.

The concentrations of ions in the dispersion medium were determined spectrophotometrically for liposome dispersions untreated and treated with PEF. For comparison, analogous studies were carried out for yeast cells. The results are shown in Figure 5 and Figure 6.

Specifically, in the liposome dispersion without PEF treatment, divalent cations (Ca^2+^ and Mg^2+^) are removed from the bulk phase through adsorption onto the polar headgroups of POPC (phosphatidylcholine) liposome membranes. Initially, the loss of Ca^2+^ concentration was 2.8 μg/mL, and that of Mg^2+^ was 2.2 μg/mL. Increasing the concentration of POPC enhanced the available binding sites, resulting in higher decline levels of 4.0 μg/mL for Ca^2+^ and 3.3 μg/mL for Mg^2+^. This baseline ion loss from the dispersion medium was observed to increase liposome size, surface charge, and dispersion stability.

PEF significantly enhanced ion uptake by inducing electroporation. This process created transient pores in the lipid bilayer, allowing ions to pass through and accumulate. The most rapid decrease in ion concentration occurred immediately after PEF application. Due to the higher conductivity of the Mg^2+^ medium, the specific energy input reached 43.2 kJ/kg, which could suggest more efficient membrane electroporation compared to the Ca^2+^ medium treated at 28.8 kJ/kg. However, observed changes in the medium’s ion concentration indicate a greater loss of Ca^2+^ than Mg^2+^ ions, implying a higher Ca^2+^ accumulation in liposomes (Figure 5) and yeast cells (Figure 6). Two hours post-PEF, the total Ca^2+^ concentration was found to have decreased by 3.2-fold and Mg^2+^ decreased by 2.5-fold, irrespective of the initial POPC concentration. These changes correspond to an ion concentration decline in the medium of 9% and 13% for Ca^2+^, and 5.7% and 8.7% for Mg^2+^, at lower and higher POPC concentrations, respectively. These findings suggest that specific energy contribution is not the dominant factor of ion influx. Instead, the physicochemical properties of the ions, including their degree of hydration, affinity for the membrane, and specific ion-membrane interactions, play a more significant role. This enhanced uptake led to further increases in liposome size, charge, and dispersion stability. Further changes observed after 1–2 days are likely attributed to subsequent dispersion instability and liposome aggregation.

In addition to pore formation, another mechanism for ion absorption can be considered, assuming that the applied field parameters are insufficient for electroporation of nanometer-sized liposomes. This mechanism exploits the chemical properties of divalent cations and their interaction with the electric field to bypass the necessary condition of high-voltage for pore-forming electroporation. PEF induces a momentary, high local concentration of positively charged Ca^2+^ and Mg^2+^ ions towards the liposome surface. These cations can then bind strongly to the negatively charged phosphate groups of POPC, acting as bridges between adjacent lipid molecules. This binding reduces electrostatic repulsion and promotes denser lipid packing, which in turn can induce local structural defects or lead to transient phase states that favor pore formation on the liposome bilayer surface [51,52]. In addition, instead of typical aqueous pores, the stress-induced defects provide the pathway for the small ions to rapidly partition into the membrane or cross into the lumen, explaining the measured total ion uptake without the high transmembrane potential necessary for conventional electroporation. Moreover, ion penetration via both mechanisms is also possible and the visible changes may be the result of both effects.

Figure 6 shows the changes in the concentrations of Ca^2+^ and Mg^2+^ ions in the yeast cell suspension before and immediately following a 600 s PEF treatment. Exposure to PEF promoted higher permeability of the cell membrane towards the investigated ions, consequently lowering their concentration in the solution by nearly 13% for Ca^2+^ and 11% for Mg^2+^. On this basis, it can be concluded that the selection of appropriate field parameters and operating time is crucial for various applications and can be quite smoothly adjusted depending on specific needs.

Ions can be transported across the cell membrane either by active mechanisms involving specific channels and pumps or by passive processes that transiently enhance membrane permeability. Despite extensive studies, the precise molecular basis of passive ion transport remains unresolved because of the complexity of membrane structures. Proposed mechanisms include thermal pore formation, solubility-diffusion, lipid flip-flop, and ion-induced pore formation, yet it is still unclear which mechanism predominates.

Ca^2+^ and Mg^2+^ ions enter cells exposed to PEF mainly through electroporation-induced pores in the membrane, aided by diffusion and electrophoretic forces. The extent of ion transport depends on pulse strength, duration, and number [53]. According to González-Cuevas et al. [6] the main driving force of ionic uptake during the electric pulse is the one due to the externally applied electric field (electrophoretically mediated transfer). In addition, diffusivity (passive transport) of ions depends among others on their effective size (hydrated radius) [54]. According to data in Table 1, an effective ionic radius of Mg^2+^ is slightly bigger than Ca^2+^ which may explain the minor difference in ion concentration in the solution after PEF exposure.

These results align with data obtained for liposomes after the same time of PEF action (discussed earlier), which suggests a similar underlying mechanism of membrane response. The authors recognize that the liposomes used represent a simplified model of the cell. However, liposome composition can be modified quite easily, suggesting the possibility of bringing this model closer to the real cell by increasing the complexity of the liposomes in the future. Moreover, combining liposomes and yeast in studies and attempting to identify similarities in their behavior is one way to achieve a more accurate representation of real-world systems. These indirect conclusions represent another step towards a more comprehensive understanding of the mechanisms of the processes being studied and a solid basis for future planned research.

## 3. Materials and Methods

### 3.1. Materials

1-Palmitoyl-2-oleoyl-*sn*-glycero-3-phosphatidylcholine (POPC) was purchased from Sigma-Aldrich (St. Louis, MO, USA), chloroform (CHCl_3_) and calcium chloride dehydrate (CaCl_2_ × 2H_2_O) from Chempur (Piekary Śląskie, Poland), methanol (CH_3_OH) from Romil Pure Chemistry (Cambridge, UK), and magnesium chloride hexahydrate (MgCl_2_ × 6H_2_O) from Avantor Performance Materials Poland S.A. (Gliwice, Poland). All reagents were of high purity (≥99.0%). The industrial strain of *S. cerevisiae* 11 B1 from the Yeast Plant (Lublin, Poland) was used for the experiments. A 1 or 2 mg/mL POPC solution was prepared using chloroform as a solvent. Ultrapure Milli-Q water, Merck KGaA, Darmstadt, Germany (resistivity of 18.2 MΩcm, surface tension of 72.0 mN/m at 25 °C, and a pH of 5.6) served as the subphase for Langmuir monolayer experiments and for the preparation of all aqueous solutions. The final electrolyte solutions were prepared to contain cations at a concentration of 100 μg/mL.

### 3.2. Methods

#### 3.2.1. Compression Isotherms

All monolayer experiments were conducted at a constant temperature of 25 °C using a thermostated Langmuir-Blodgett trough (LB KSV NIMA trough, Biolin Scientific, Stockholm, Sweden) and followed a previously published procedure [26]. The trough was placed in the laser safety cabinet and isolated from surrounding vibrations by the active vibration isolation system. A 70 μL chloroform solution of POPC (1 mg/mL) was deposited onto an aqueous subphase containing Ca^2+^ or Mg^2+^ ions (100 μg/mL). After the solvent evaporated, the resulting POPC monolayer was symmetrically compressed at a constant rate of 10 mm/min until it collapsed. The surface pressure changes were obtained as a function of the mean area per molecule (π−A isotherms).

The π−A isotherm data was used to calculate the compression modulus ($C_{S}^{-1}$) which is a measure of how resistant a monolayer is to being compressed. This was accomplished by numerically computing the first derivative according to Equation (1):

(1) $$C_{S}^{-1}=-A{\left(\frac{d\pi }{dA}\right)}_{p,T}$$

where A represents the area per molecule corresponding to the measured surface pressure π, all at constant external pressure (p) and temperature (T).

#### 3.2.2. Monolayer Thickness

Simultaneously, a Brewster angle microscope, BAM (nanofilm_ultrabam, Accurion GmbH, Göttingen, Germany) coupled to an LB KSV NIMA trough (Biolin Scientific) was used to observe the morphology and to determine the thickness of the monolayers at the liquid-air interface. The BAM system used a 50 mW solid-state laser emitting light at a 658 nm wavelength. The laser initially produced purely *p*-polarized light (linearly polarized in the plane of incidence, or vertically polarized). To ensure high quality, the light passed through a polarizer to significantly reduce any non-polarized component, aiming for a polarization ratio (non-polarized light/total light) of 10^−8^. When this highly *p*-polarized light struck the pure water surface at the Brewster angle (approximately 53.2°), almost no light was reflected. This resulted in a dark background with no image contrast. Reflection occurred, and contrast was generated, when a monolayer with a refractive index different from water’s (1.33) was spread onto the surface. This reflected light then traveled through an objective lens and an analyzer before hitting a CCD camera (Accurion GmbH, Göttingen, Germany) (1392 × 1040 pixels, max 40 frames per second). These optics and the camera were positioned to capture the reflected beam, allowing for the acquisition of high-quality, high-contrast images of the monolayer’s lateral structure.

To find the relative thickness (d) of the film in each measurement, a two-step process was used: camera calibration followed by applying a single-layer optical model. First, the camera was calibrated (following the method by Rodriguez Patino et al. [55]). This step established the relationship between the measured grey level (the digital intensity value from the camera) and the relative reflectivity (R) of the light. This involved generating a plot of grey level versus the angle of incidence, which was then fitted to a parabola. The minimum point of this parabolic fit indicated the angle of incidence that resulted in the lowest reflectivity under the specific experimental conditions. The resulting calibration factor allowed to accurately convert the greyscale data from the images directly into values of reflectivity (R).

Once the reflectivity (R) was known, the single-layer optical model (Equation (2)) (from Winsel et al. [56]) was applied to convert the reflectivity value into the film thickness (d).

(2) $$R=\frac{I_{r}}{I_{0}}={\left(\pi \frac{d}{\lambda }\right)}^{2}\frac{{\left(n_{1}^{2}-n_{2}^{2}-1+\frac{n_{2}^{2}}{n_{1}^{2}}\right)}^{2}}{1+n_{2}^{2}}$$

where R—reflectivity, which is the ratio of the reflected intensity ($I_{r}$) to the incident intensity ($I_{0}$), $n_{1}$—refractive index of the monolayer, $n_{2}$—refractive index of the subphase, λ—wavelength of the incident laser light.

#### 3.2.3. Adsorption Isotherms

Monolayer stability was assessed using adsorption isotherms. For this purpose, the surface area per molecule in a POPC monolayer on a subphase containing Ca^2+^ or Mg^2+^ ions was determined as a function of time. The experiment was conducted at a constant surface pressure of 30 mN/m, which was maintained by oscillatory barrier movements at a rate of 5 mm/min. This surface pressure value was chosen because it corresponds to the internal lateral pressure of natural biological membranes, thereby ensuring a similar degree of molecular packing [28].

#### 3.2.4. Preparation of Liposome Dispersions

Liposomes were obtained by hydrating a dry film of the phospholipid POPC [57]. First, POPC solutions were prepared in chloroform at concentrations of 1 mg/mL and 2 mg/mL. After the solvent was evaporated in a vacuum dryer (117 mbar), the resulting lipid film was dispersed in Milli-Q water in an ultrasonic bath (10 min) to achieve final lipid concentrations of 0.05 mg/mL and 0.1 mg/mL. To standardize the liposome dimensions, the dispersion was passed repeatedly (15 times) through a 0.2 μm polycarbonate membrane using a hand extruder (Avanti Polar Lipids, Inc., Alabaster, AL, USA). Following this, a calcium or magnesium chloride solution was added to obtain a final cation (Ca^2+^ or Mg^2+^) concentration of 100 μg/mL. For the control samples (“water”), the salt solution was replaced with an equivalent volume of Milli-Q water. The resulting dispersions were stored in a refrigerator before further testing. The liposome characterization involved the preparation of two independent series of liposome dispersions for each experiment.

#### 3.2.5. Yeast Cultivation

Yeast cultivation was performed based on the protocol of Pankiewicz et al. [7]. Briefly, cells were grown in a 500 mL flask using a culture medium with a pH of 6, which contained yeast extract (10 g/L), peptone (20 g/L), and glucose (20 g/L). Under sterile conditions, the contents of a single agar slant were transferred to 150 mL of this medium. The flask was then placed in a continuously stirred incubator (150 rpm) and grown for 24 h at 30 °C. After cultivation, the flask was refrigerated for another 24 h. The medium was then decanted, and the resulting sediment was suspended in 100 mL of physiological saline before being stored at 4 °C. The content of lipids in yeast was 2.70 ± 0.14% (cell dry weight) [42]. Accordingly, the lipid concentration in the electroporated sample with yeast was about 0.0162 mg/mL. Increasing the yeast biomass to match the lipid content of the liposome samples would require approximately 1.85–3.70 mg of dry cells per 0.5 mL, corresponding to about 7.4 × 10^7^ to 1.48 × 10^8^ cells/mL. At such concentrations, yeast suspensions are known to undergo significant aggregation, particularly in the presence of Mg^2+^ or Ca^2+^ ions, leading to heterogeneous particle distribution [58]. This effect would compromise the uniformity of the electric field during PEF treatment and result in uncontrolled variability in the effective energy delivered to individual cells. Consequently, the lipid concentration in the yeast sample could not be matched to that of the liposome dispersions without negatively affecting the integrity and reproducibility of the PEF experiments.

#### 3.2.6. Optical Density Measurements

To determine the number of yeast cells in the suspension, the optical density (OD) of the sample was measured spectrophotometrically (Cary 50 Scan, Varian Inc., Palo Alto, CA, USA) at a wavelength of 600 nm [10]. Using a standard curve, the number of cells in the sample was estimated to be 6 × 10^6^ which promotes fairly uniform electroporation.

#### 3.2.7. Stability of Liposome Dispersions

Liposome stability measurements were conducted at 25 °C, at three distinct time points: immediately after dispersion preparation, and after 24 and 48 h. The size distribution (mean diameter and polydispersity index) and zeta potential of the POPC liposome dispersion were characterized using a Zetasizer Nano-ZS (Malvern Instruments, Malvern, UK) with a 633 nm laser set at a 173° angle. These parameters were determined via dynamic light scattering (DLS) and laser Doppler microelectrophoresis (LDE), respectively. Sizes were quoted as the z-average mean for the liposomal hydrodynamic diameter. The zeta potential was calculated based on electrophoretic mobility of particles using the Smoluchowski equation (Equation (3)):

(3) $$u_{e}=\frac{\varepsilon \zeta }{\eta }f(\kappa a)$$

where $u_{e}$—electrophoretic mobility, ε—permittivity of the medium, η—viscosity, ζ—zeta potential, f(κa)—Henry’s function dependent on the bulk conductivity.

Measurements of liposome size (via DLS), electrophoretic mobility and conductivity (for zeta potential calculations) were performed in triplicate for each liposome dispersion. Each reported mean value was calculated by averaging data from 3 to 6 individual measurements and is presented with the standard deviation (±SD). To ensure high data quality, each individual measurement incorporated averaging across 10 to 100 runs (or acquisitions). The “Automatic” setting of the Zetasizer software (v8.02) was utilized, allowing the instrument to optimally determine the number and duration of these runs within the 10–100 range. The optimization was crucial to ensure that the measurement captured a reliable and stable average of the scattered light fluctuations, which reflects the Brownian motion of the particles. This process is necessary for accurately determining the decay of the intensity autocorrelation function across all relevant time scales, thereby leading to stable and accurate liposome size and electrophoretic mobility/zeta potential results.

Time-stable dispersions were obtained. Those aged 24 h were subsequently utilized for further studies on ion absorption into liposomes under a pulsed electric field (PEF).

#### 3.2.8. Electroporation of Samples

Liposomal dispersions fabricated by dry film hydration and yeast cells (centrifuged from physiological saline and washed once with Milli-Q water) were electroporated in cuvettes equipped with aluminum electrodes. A sample of liposomes or yeast, suspended in a solution containing 100 μg/mL Ca^2+^ or Mg^2+^ ions, respectively, was placed between the electrodes (gap width of 0.2 cm). Control samples (“water”) consisted of liposome dispersions in Milli-Q water were treated in the same way as samples containing Ca^2+^ or Mg^2+^ ions. The total volume of the sample was 0.5 mL. The cuvette was then inserted into a BTX attachment (Safety stand, model 630 B, BTX Harvard Apparatus, Holliston, MA, USA), and a unipolar square-wave generator ECM 830 (BTX Harvard Apparatus, Holliston, MA, USA) was activated. The following parameters were used: field strength of 2 kV/cm, pulse width of 20 μs, pulse frequency of 1 Hz, and time of treatment of 600 s. The PEF parameters were chosen based on existing literature on the optimization of pulsed electric field exposure for the yeast *Saccharomyces cerevisiae* [8]. These specific parameters were found to be the most effective at maximizing ion accumulation within the cells. The value of specific energy input was calculated according to the formula [59]:

(4) $$W=\frac{\sigma \cdot E^{2}\cdot r\cdot n}{\rho }$$

where *σ*—conductivity (S/m), *E*—electric field (V/m), *r*—pulse duration (s), *n*—number of pulses, ρ—density (kg/m^3^).

The specific energy input was 28.8 kJ/kg and 43.2 kJ/kg for solution containing Ca^2+^ and Mg^2+^ ions, respectively. The corresponding temperature increase in the 0.5 mL electroporated solution was negligible (ΔT < 0.1 °C).

After the PEF treatment, the dispersion was transferred to an Eppendorf tube and centrifuged at 4000 rpm for 5 min. Following centrifugation, 0.2 mL of the supernatant was collected, and the concentration of Ca^2+^ or Mg^2+^ ions was determined colorimetrically (described below).

#### 3.2.9. Spectrophotometric Measurements

The concentration of the ions that remained in the solution was determined spectrophotometrically using the Liquick-Cor-CALCIUM ARSENAZO diagnostic kit for Ca^2+^ ions and the Liquick-Cor-MG kit for Mg^2+^ ions (Cormay Diagnostics S.A., Łomianki, Poland). Absorbance measurements were performed on a Cecil CE 1011 spectrophotometer (Cecil Instruments Ltd., Cambridge, UK) at a wavelength of 650 nm for Ca^2+^ and 520 nm for Mg^2+^. For Ca^2+^, the ions formed a blue complex with arsenazo III dye at a neutral pH. For Mg^2+^, the ions reacted with xylidyl blue in an alkaline environment to form a purple-colored compound. In both cases, the resulting color intensity is directly proportional to the ion concentration in the sample. The absorbance of the standard and test samples was read against a reagent blank, which allowed for the calculation of the concentration of Ca^2+^ and Mg^2+^ ions (μg/mL) that did not penetrate the liposomes or yeast cell membrane. The amount of ions accumulated in the liposomes or yeast cells was determined from the difference in the concentration of the starting solution with suspended liposomes or yeast and the solution after PEF treatment. For liposomes, analysis of concentration changes was performed over time, specifically: immediately after PEF exposure, and at 2 h, 24 h, and 48 h, compared to samples without PEF.

#### 3.2.10. Statistical Analysis

All data were expressed as mean ± standard deviation (SD). Statistical differences among multiple treatment groups were evaluated using a one-way analysis of variance (ANOVA). When the ANOVA indicated significant differences (*p* < 0.05), pairwise comparisons were performed using Tukey’s Honestly Significant Difference (HSD) post hoc test at α = 0.05. The statistical analyses were conducted separately for each liposome concentration (0.05 and 0.1 mg/mL) in all tested media. Within each concentration, all experimental groups presented in a given figure were included in the same statistical model and compared simultaneously. Control samples, in which liposomes were dispersed in Milli-Q water and treated in the same manner as ion-containing samples, were included as regular groups in the analysis. The results of Tukey’s post hoc test are presented using a letter-based notation above the bars in the figures. Groups sharing at least one letter are not significantly different, whereas groups with different letters differ significantly. Statistical analyses were performed using Excel software (Microsoft, Redmond, WA, USA).

## 4. Conclusions

The enrichment of microorganisms with essential elements and microelements is a primary objective in the food industry and human nutrition, as it contributes to the production of nutritionally superior foods. This study was therefore designed to examine how pulsed electric field influences the penetration of Ca^2+^ and Mg^2+^ ions into liposomes—phospholipid vesicles serving as a simplified model for the cell membranes of the industrially important yeast, *Saccharomyces cerevisiae*. The results show significant differences in the behaviour of the ions used. Divalent cations collectively alter the physicochemical properties of the membranes investigated, as shown by the surface pressure versus mean molecular area isotherms. These modifications to the model membrane’s material properties can be attributed to two competing effects. First, electrostatic screening of the POPC headgroups by hydrated Mg^2+^ leads to film expansion due to increased repulsion between the lipid-ion pairs. Second, a condensation effect is driven by the bridging of divalent Ca^2+^ cations to the phosphate groups in the lipid headgroups, connecting adjacent lipid molecules.

By expanding our physicochemical studies on model cell membranes, we were able to indirectly compare them with natural cell systems and correlate their properties. The results demonstrate notable differences at the molecular level in how the selected cations interact with the phospholipid monolayer. The affinity of Ca^2+^ ions for the model POPC membranes is slightly greater than that of Mg^2+^ ions. Both cations penetrate deep into the hydrophilic region of the membrane, but due to their various modes of interaction, they differently influence the degree of hydration of the membrane containing the absorbed ions. This results in observable differences in liposome dispersion stability in the presence of these cations. The processes of vesicle aggregation and fusion appear to be closely related to the ability of the studied ions to interact with the polar groups of POPC and release solvation water molecules.

Pulsed electric field exposure was found to increase the concentration of ions absorbed by liposomes, which in turn changes their size distribution and surface charge/electrokinetic potential. This modification was attributed to the interaction of cations with the negatively charged sites on the polar heads of lipids, thus masking these sites at the liposomes’ surface. By using appropriately selected electric field parameters, the accumulation of cations in the model membranes relatively well correlated with the increased Ca^2+^ and Mg^2+^ content observed in real yeast cells. Both liposomes and yeast cells accumulated approximately 6–13% more Ca^2+^ and Mg^2+^ ions when compared to the control sample.

Our analysis of the penetration and incorporation of inorganic ions has led to a better understanding of the phenomena at the cell membrane-environment interface. This new acquired knowledge provides a solid basis for future research into encapsulating and releasing various active substances using cells or liposome carriers under precise conditions.

## Figures and Tables

**Table 1:** Tabulated chemical properties of Ca^2+^ and Mg^2+^ ions.

Chemical Property	Ca^2+^	Mg^2+^	References
Ionic radii (Å)	0.99	0.65	[23]
	1.00	0.75	[21]
	1.00	0.72	[25]
Hydrated radii (Å)	4.19	4.28	[23]
Solvation energy (kcal/mol)	−354.7	−433.3	[23]
Electronegativity	1.00	1.20	[23]

**Table 2:** Langmuir monolayer characterizing parameters based on representative compression isotherms presented in Figure 1.

POPC Monolayer	$A_{0}$ [Å^2^/molec.]	$\pi_{C}$ [mN/m]	$C_{S,30}^{-1}{/C}_{S,max}^{-1}$ [mN/m]	$A_{lim}$ [Å^2^/molec.]	D [Å]	$d_{30}$$/d_{max}$ [nm]
water	111.7	41.7	97.7/105.0	61.6	8.86	2.73/2.90
Ca^2+^	108.9	42.9	97.8/110.6	60.3	8.76	3.09/3.26
Mg^2+^	114.5	41.4	96.3/100.6	63.9	9.02	2.29/2.49

**Table 3:** Polydispersity index (PDI) ± SD for liposome dispersions in all tested media with a total POPC concentration of 0.05 mg/mL. Statistical analysis was performed using one-way ANOVA followed by Tukey’s HSD post hoc test; all values presented in the table were included in the same statistical comparison. Means sharing at least one letter are not significantly different, whereas values with different letters differ significantly (*p* < 0.05).

Time [h]	Polydispersity Index (PDI) ± Standard Deviation for 0.05 mg/mL Liposome Dispersions		
	in Water	in Ca^2+^ Solution	in Mg^2+^ Solution
without PEF			
0	0.198 ± 0.028 c	0.093 ± 0.009 a	0.098 ± 0.013 a
24	0.172 ± 0.005 bc	0.121 ± 0.015 a	0.103 ± 0.004 a
48	0.226 ± 0.007 c	0.159 ± 0.008 b	0.142 ± 0.016 a
with PEF			
0	0.172 ± 0.005 bc	0.202 ± 0.025 c	0.240 ± 0.018 cd
2	0.164 ± 0.016 b	0.205 ± 0.015 c	0.310 ± 0.058 d
24	0.166 ± 0.013 b	0.220 ± 0.017 c	0.640 ± 0.042 f
48	0.172 ± 0.023 bc	0.394 ± 0.027 e	0.670 ± 0.078 f

**Table 4:** Polydispersity index (PDI) ± SD for liposome dispersions in all tested media with a total POPC concentration of 0.1 mg/mL. Statistical analysis was performed using one-way ANOVA followed by Tukey’s HSD post hoc test; all values presented in the table were included in the same statistical comparison. Means sharing at least one letter are not significantly different, whereas values with different letters differ significantly (*p* < 0.05).

Time [h]	Polydispersity Index (PDI) ± Standard Deviation for 0.1 mg/mL Liposome Dispersions		
	in Water	in Ca^2+^ Solution	in Mg^2+^ Solution
without PEF			
0	0.141 ± 0.020 b	0.067 ± 0.005 a	0.061 ± 0.002 a
24	0.158 ± 0.019 b	0.132 ± 0.013 b	0.099 ± 0.013 a
48	0.165 ± 0.006 bc	0.078 ± 0.011 a	0.146 ± 0.040 b
with PEF			
0	0.150 ± 0.023 b	0.160 ± 0.002 b	0.111 ± 0.009 a
2	0.140 ± 0.020 b	0.165 ± 0.015 bc	0.140 ± 0.016 b
24	0.175 ± 0.025 c	0.169 ± 0.011 bc	0.222 ± 0.012 d
48	0.160 ± 0.012 b	0.173 ± 0.020 c	0.280 ± 0.023 e

## Data Availability

Data are contained within the article.

## Supplementary Materials

The following supporting information can be downloaded at: https://www.mdpi.com/article/10.3390/molecules31010151/s1, Figure S1: Compression isotherms of POPC monolayer on: (**a**) a water subphase, (**b**) in the presence of Ca^2+^ ions, (**c**) in the presence of Mg^2+^ ions, registered at 25°C; Figure S2: Time-dependent changes in the conductivity of POPC liposome dispersions in water, Ca^2+^ or Mg^2+^ solutions, (**a**) 0.05 mg/mL and (**b**) 0.1 mg/mL, as determined by microelectrophoresis. The conductivity of medium is also included for comparison. Error bars denote ± SD. Statistical analysis was performed using one-way ANOVA followed by Tukey’s HSD post hoc test. Different letters above the bars indicate statistically significant differences within each liposome concentration among all tested media and time points (*p* < 0.05); Figure S3: Time-dependent changes in the electrophoretic mobility of POPC liposome dispersions in water, Ca^2+^ or Mg^2+^ solutions, (**a**) 0.05 mg/mL and (**b**) 0.1 mg/mL, as determined by microelectrophoresis. The electrophoretic mobility of the medium is also included for comparison. Error bars denote ± SD. Statistical analysis was performed using one-way ANOVA followed by Tukey’s HSD post hoc test. Different letters above the bars indicate statistically significant differences within each liposome concentration among all tested media and time points (*p* < 0.05).
