# Supplementary material for: Permeability of Phospholipid Membranes to Divalent Cations: The Effect of Pulsed Electric Field
Source: Molecules. 2026 Jan 1;31(1):151. doi: 10.3390/molecules31010151 (PMC12788142; doi:10.3390/molecules31010151)
Supplement: Supplementary file 1 [file molecules-31-00151-s001.zip › molecules-4004108-supplementary.pdf]

S2. Results and Discussion

S2.1. Langmuir monolayers

All compression isotherms acquired for the POPC monolayer on different subphases are shown in a supplementary figure (Figure S1). The representative isotherms are presented and discussed in the main text.

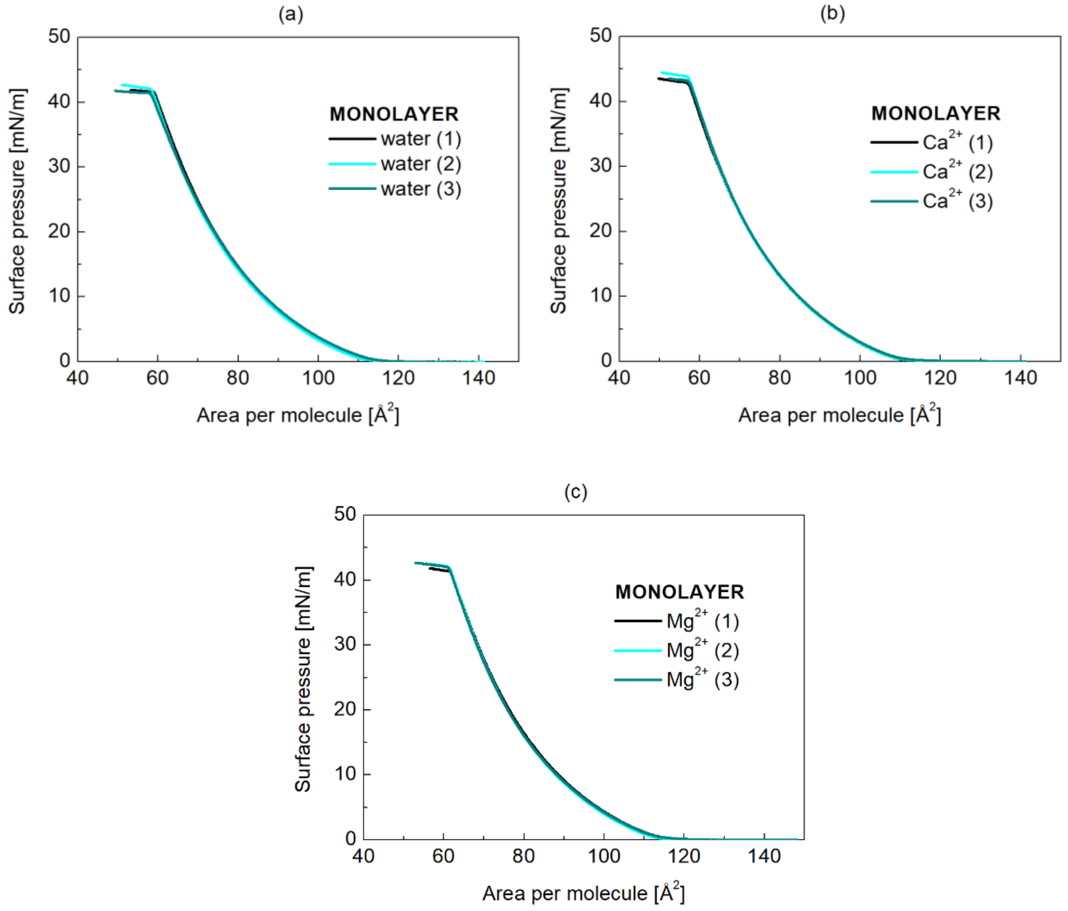

Figure S1. Compression isotherms of POPC monolayer on: (**a**) a water subphase, (**b**) in the presence of Ca^{2+} ions, (**c**) in the presence of Mg^{2+} ions, registered at 25°C.

S2.2. Liposomes

The time dependence of both conductivity and electrophoretic mobility for POPC liposomes, before and after PEF treatment in different media, is shown in Figures S2 and S3, respectively. An adequate explanation is provided in the main text.

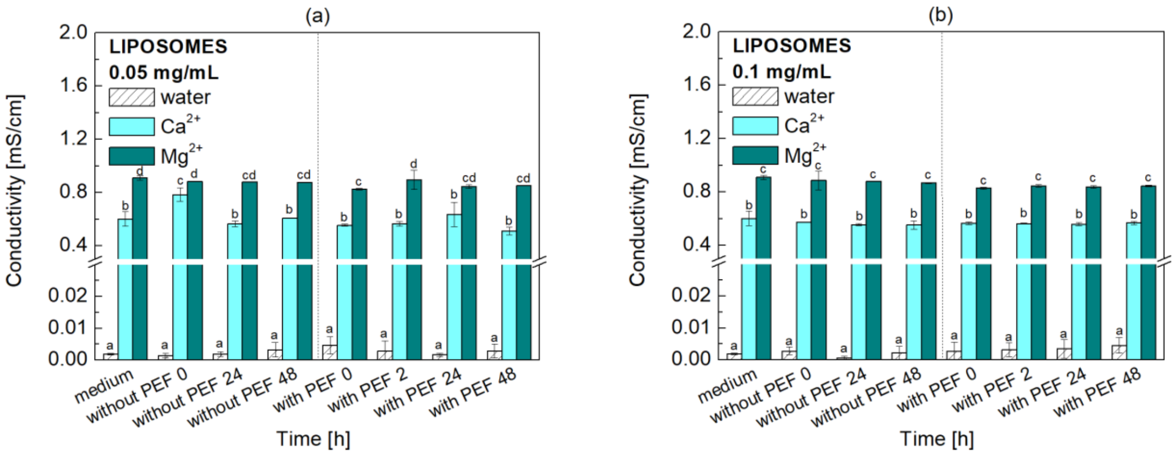

Figure S2. Time-dependent changes in the conductivity of POPC liposome dispersions in water, Ca^{2+} or Mg^{2+} solutions, (**a**) 0.05 mg/mL and (**b**) 0.1 mg/mL, as determined by microelectrophoresis. The conductivity of medium is also included for comparison. Error bars denote ± SD. Statistical analysis was performed using one-way ANOVA followed by Tukey's HSD post hoc test. Different letters above the bars indicate statistically significant differences within each liposome concentration among all tested media and time points ($p < 0.05$).

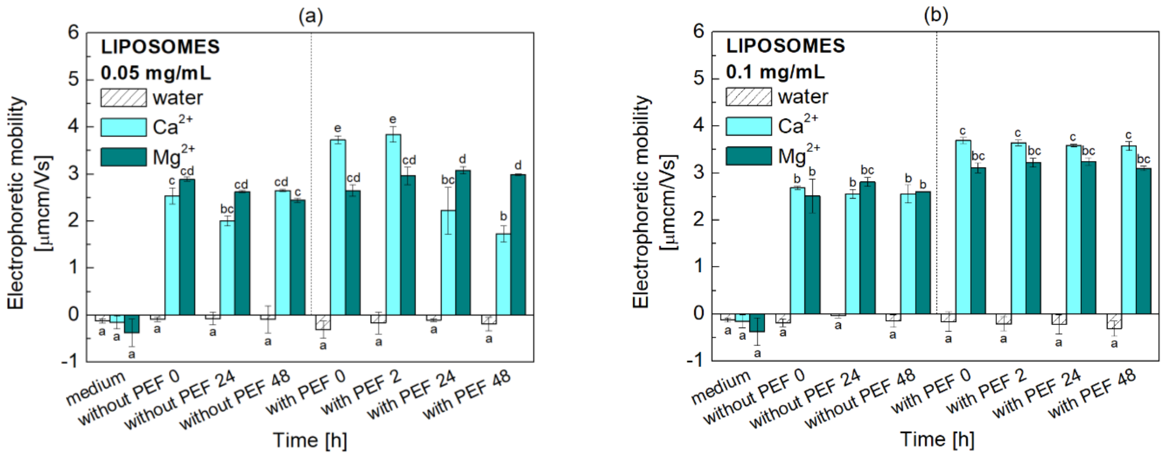

Figure S3. Time-dependent changes in the electrophoretic mobility of POPC liposome dispersions in water, Ca^{2+} or Mg^{2+} solutions, (**a**) 0.05 mg/mL and (**b**) 0.1 mg/mL, as determined by microelectrophoresis. The electrophoretic mobility of the medium is also included for comparison. Error bars denote ± SD. Statistical analysis was performed using one-way ANOVA followed by Tukey's HSD post hoc test. Different letters above the bars indicate statistically significant differences within each liposome concentration among all tested media and time points ($p < 0.05$).
